# Comparative efficacy of combined CTLA-4 and PD-1 blockade vs. PD-1 monotherapy in metastatic melanoma: a real-world study

**DOI:** 10.1038/s44276-024-00041-1

**Published:** 2024-02-13

**Authors:** Avital Klein-Brill, Shlomit Amar-Farkash, Keren Rosenberg-Katz, Ronen Brenner, Jürgen C. Becker, Dvir Aran

**Affiliations:** 1 Carelon Digital Platforms, Tel Aviv, Israel; 2 Department of Oncology, Edith Wolfson Medical Center, Holon, Israel; 3 Department of Dermatology, University Hospital Essen, Essen, Germany; 4 Translational Skin Cancer Research, German Cancer Consortium (DKTK), Partner Site Essen, Medical Faculty, University of Duisburg-Essen, Essen, Germany; 5 German Cancer Consortium (DKTK), University of Duisburg-Essen, Essen, Germany; 6 German Cancer Research Center (DKFZ), Heidelberg, Germany; 7 Faculty of Biology, Technion-Israel Institute of Technology, Haifa, Israel; 8 The Taub Faculty of Computer Science, Technion-Israel Institute of Technology, Haifa, Israel

## Abstract

**Background:**

In light of the substantial toxicity associated with combined CTLA-4 and PD-1 blockade (ipilimumab and nivolumab), we assessed its efficacy and safety against anti-PD-1 monotherapy (nivolumab or pembrolizumab) in patients with metastatic melanoma under real-world conditions.

**Methods:**

We conducted a retrospective observational study involving 962 patients with stage IV metastatic melanoma who initiated adjuvant treatment between January 2017 and December 2021 across outpatient clinics in the United States. We adjusted for variables such as age, sex, ECOG performance status, comorbidity index, social deprivation index, metastatic sites, BRAF mutation status, and year of treatment. Outcomes included overall survival (OS) and post-treatment hospitalizations, analyzed using propensity score adjustment and inverse probability of treatment weighted Kaplan–Meier estimators.

**Results:**

After adjusting for all variables, no significant difference in OS was observed between treatment protocols in the overall cohort (*P* = 0.417). In patients with multi-organ metastasis (involvement of more than two organ systems), combined CTLA-4 and PD-1 blockade was associated with improved OS (*P* = 0.033). Conversely, monotherapy yielded significantly better OS in patients with oligo-organ metastasis (involvement of two or fewer organ systems; *P* = 0.008). Patients with oligo-organ metastasis also experienced higher hospitalization rates due to immune-related adverse events when treated with combination therapy (31.2% vs. 8.5%, *P* < 0.001).

**Conclusions:**

Our real-world data indicate that combined CTLA-4 and PD-1 blockade is most beneficial for patients with multi-organ metastasis, while those with oligo-organ metastasis fare better with PD-1 monotherapy. The underlying reasons for these observations—whether they are due to differences in the characteristics of multi- and oligo-metastatic melanomas or the risk-benefit profile of the therapies—remain to be elucidated. These findings underscore the need for a nuanced approach to treatment regimens for stage IV melanoma patients.

## Background

Melanoma is a highly aggressive form of cancer that primarily originates from cutaneous melanocytes and, to a lesser extent, mucous membranes. The introduction of immune checkpoint inhibitors (ICIs) has revolutionized the treatment landscape, significantly extending overall survival (OS) while often maintaining a good quality of life for patients. The FDA has approved various ICIs for advanced melanoma, including the CTLA-4 inhibitor ipilimumab and PD-1 inhibitors such as nivolumab and pembrolizumab.

Head-to-head clinical trials like KEYNOTE-006 [1] and CheckMate-067 [2] have demonstrated a clear survival advantage for PD-1 inhibitors over CTLA-4 inhibitors, with three-year OS rates exceeding 50%. The CheckMate-067 trial also evaluated the combination of ipilimumab and nivolumab, revealing a modest but statistically insignificant survival benefit over nivolumab monotherapy at three years (58% vs. 52%). This benefit persisted in long-term follow-up analyses at five and 6.5 years [3, 4].

Combination therapy has become the standard of care for specific patient populations, such as those with asymptomatic central nervous system (CNS) metastases, BRAF mutations, and elevated serum LDH levels [5]. Currently, over a third of stage IV melanoma patients receive this combination therapy. However, it comes with a higher risk of immune-related adverse events (irAEs), with 59% of patients experiencing grade 3-4 irAEs compared to 21% on monotherapy [2]. Subsequent studies and clinical experience have corroborated this elevated toxicity [6]. Furthermore, the full spectrum of its toxicity is not well known even today, and new types of side effects continue to be reported [7, 8].

While randomized clinical trials are the gold standard for evidence-based medicine, their findings may not always be generalizable to real-world patient populations. Several retrospective studies have confirmed the general efficacy of ICIs in real-world settings [9, 10]. However, the survival benefit of combination therapy that was overserved in the clinical trial have not been shown in real-world setting. A meta-analysis of randomized clinical trials from non-melanoma tumors found no survival benefit for the combination therapy [11]. Given the marginal survival benefit and high toxicity associated with combination therapy, further investigation is warranted in melanoma as well.

To address these gaps, we analyzed a comprehensive dataset of 962 patients with advanced melanoma who received ICI treatment across various U.S. healthcare settings. Our study aims to identify patient and disease characteristics that may influence the differential benefit of monotherapy versus combination therapy, thereby contributing to the advancement of precision medicine.

## Methods

### Data source

We conducted a retrospective study using national clinical data from the Carelon Cancer Care Quality Program (CCQP), supplemented with associated claims and laboratory data. The dataset includes treatment regimens, AJCC staging, ECOG performance status, ICD-10 diagnosis codes, and BRAF status, among other variables. The claims data cover approximately 45 million commercially insured members but exclude government, Medicare, and Medicaid claims. All data were anonymized to protect patient confidentiality. Death was inferred from discharge status and supplemented with national death registries and obituary data.

### Cohort definition

Our cohort comprised 962 patients diagnosed with stage IV cutaneous melanoma between January 2017 and December 2021. Inclusion criteria were initiation of first-line treatment and an ECOG performance score between 0 and 2. Patients were followed until July 2023 or death, whichever occurred first. All patients were treated in the adjuvant setting. We employed an analytical pipeline similar to that described by Klein-Brill et al. [12]. Patients were excluded if treatment plans in the CCQP clinical data did not align with claims data. Initiation of treatment (index date) was adjusted to the date of first treatment as indicated by the claims. Patients with an earlier ICI treatment according to the claims were excluded if there were more than 35 days between such early cycles, indicating possible earlier line of treatment. Our cohort spanned 432 medical oncology clinics and outpatient centers across 43 U.S. states.

### Covariates and outcomes

The primary endpoint was overall survival, defined as the time from initiation of treatment to death or last follow-up if censored. The secondary endpoint was 3-months posttreatment hospitalization. Baseline characteristics include sociodemographic information, comorbidities, and disease characteristics, ECOG performance status, AJCC stage, metastatic sites and BRAF mutational status (Table 1). Sociodemographic information included age, sex, and social deprivation index (SDI). Comorbidities were assessed using the Charlson Comorbidity Index (CCI), excluding cancer and metastases, according to patient claims in the year prior to and up to 10 days before the index date [13]. Number and location of metastatic sites were identified using metastasis associated ICD-10 codes in the one-year prior to index date and up to one month from the index date. The count of number of involved organs was performed in this manner: one site for each of the most common metastatic sites (brain, liver, lung, bone) and additional site for all other distant sites together. BRAF status was only available for 49% of the patients since it is not required to report it for treatment approval of ICIs. We note that there were significant differences between the patients with available BRAF status compared to those without available BRAF status (Supplementary Table 2), therefore we performed sensitivity analyses of the main results for both cohorts independently. Hospitalizations were classified as irAE-related using a compendium of irAE-related ICD-10 codes [6, 14] as well as additional codes based on practice (Supplementary Data).

**Table 1:** Patients characteristics.

		Combination therapy*	Monotherapy**	*P* value
N		497	465	
Age [mean (SD)]		54.74 (10.44)	56.78 (10.73)	0.003
Male sex (%)		335 (67.4)	291 (62.6)	0.133
ECOG PS (%)	0	258 (51.9)	260 (55.9)	0.446
	1	214 (43.1)	182 (39.1)	
	2	25 (5.0)	23 (4.9)	
Treatment year (%)	2017	106 (21.3)	112 (24.1)	0.002
	2018	83 (16.7)	107 (23.0)	
	2019	86 (17.3)	95 (20.4)	
	2020	102 (20.5)	79 (17.0)	
	2021	120 (24.1)	72 (15.5)	
SDI (mean (SD))^$^		40.24 (25.53)	40.17 (25.92)	0.969
CCI (mean (SD))^§^		0.57 (0.87)	0.46 (0.77)	0.052
BRAF status (%)	MUT (%)	91 (18.3)	116 (24.9)	0.015
	WT (%)	139 (28.0)	137 (29.5)	
Metastatic site (%)	Brain (%)	185 (37.2)	120 (25.8)	<0.001
	Lung (%)	217 (43.7)	152 (32.7)	0.001
	Liver (%)	153 (30.8)	77 (16.6)	<0.001
	Bone (%)	138 (27.8)	89 (19.1)	0.002
	Other sites (%)	144 (29.0)	77 (16.6)	<0.001
No. of metastatic sites (mean (SD))		1.68 (1.25)	1.11 (1.17)	<0.001

*Ipilimumab plus Nivolumab, **Pembrolizumab or Nivolumab, ^$^social deprivation index, ^§^Charlson Comorbidity index.

The dataset used for the analyses was de-identified according to the Safe Harbor privacy principles, and did not include names, dates (only differences between dates), zip codes (only rounded SDI) or any other identifying information.

### Statistical analysis

Baseline characteristics were compared between the groups using Chi-square for categorical variables and Student’s *t* tests for continuous variables. A *p* value < 0.05 was considered statistically significant. Overall survival was defined as the interval between the initiation of first-line therapy (index date) and the date of death. Patients without a mortality event were censored at the date of their last medical claim or end of study.

All analyses presented, unless otherwise indicated are based on propensity score matching of the average treatment effect which was estimated using non-parametric covariate balancing (npcbps methods from WeightIt 0.14.2) [15]. This algorithm was used since it was the only algorithm that was able to balance all the covariates. Treatment type was defined as the dependent variable, and regressed over the potentially confounding variables (age, gender, SDI, CCI, ECOG, year of treatment, BRAF status, metastatic sites or number of organs involved). To assess survival, the Kaplan–Meier estimator was weighted by the inverse propensity score (IPTW) [16] using the svyjskm function from jskm 0.4.3. The jksm package was also used to calculate hazard ratios. The PSweight 1.1.7 was used for propensity score matching of adjusted proportions. The coxph function was used to perform Cox proportional-hazards analysis. All analyses were performed using R 4.2.0. The propensity score matched cohort was also used for the posttreatment hospitalization analysis to calculate a weighted average of the hospitalized patients. Chi-square test was used to calculate significance.

## Results

### Study population

**Fig. 1:**
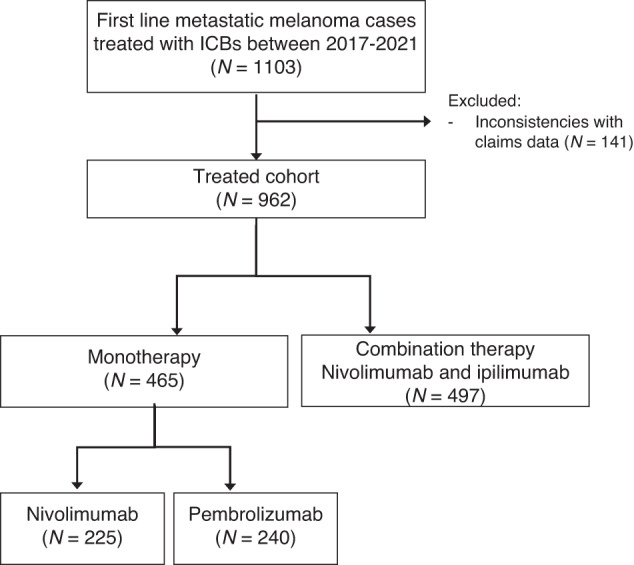
CONSORT diagram.

Using clinical data and administrative claims, we identified a cohort of 962 patients diagnosed with stage IV melanoma from January 2017 to December 2021 who were at least 18 years of age at the start of their first-line treatment. We included only patients with corroborated clinical and claims data (see “Methods” and Fig. 1). Therapy regimens were nivolumab (225 [23.4%]), pembrolizumab (240 [24.9%]) or ipilimumab plus nivolumab (497 [51.7%]). Patients treated with pembrolizumab or nivolumab had mostly similar demographics and clinical features, though nivolumab was prescribed more to males, in higher socio-demographic regions, to patients with fewer lung metastasis and slightly fewer metastatic organs involved overall (Supplementary Table 1). Importantly, we did not observe difference in OS between the monotherapy treatments (*P* = 0.412; Supplementary Fig. 1), therefore patients treated with either nivolumab or pembrolizumab were assigned to the monotherapy group, while those receiving ipilimumab plus nivolumab were assigned to the combination therapy group. Race and ethnicity were not available and were therefore not considered [17].

Comparison of the groups showed that patients receiving combination therapy differed from those treated with monotherapy in several demographic and clinical characteristics (Table 1). Patients receiving combination therapy were on average two years younger and had more metastatic organs involved. These patients also had fewer BRAF mutations. Interestingly, we observed increase in prescribing the combination therapy: from 46.7% of treatments in 2017–2019 to 59.5% in 2020–2021. The treatment decision could be predicted based on these baseline demographic and clinical features at a ROC-AUC = 0.679 (*P* < 0.001); therefore, although treatment decision appears to be partly based on clinical and demographic characteristics, it also appears to be partly stochastic or influenced by hidden variables.

Baseline characteristics of the real-world cohort varied significantly from the combination therapy cohort in the CheckMate-067 trial: our real-world patients were younger (87.1% vs. 39.2% under 65 years), had a worse performance (ECOG 0: 53.8% vs. 74.1%), and were much more likely to suffer from brain metastases (31.7% vs. 3.8%). The age difference is explained by the fact our real-world data is from commercial health insurance patients and thus excludes Medicare patients. Differences in performance status and presence of brain metastases are likely caused by the inclusion and exclusion criteria of the CheckMate-067 protocol.

### Overall survival

**Fig. 2:**
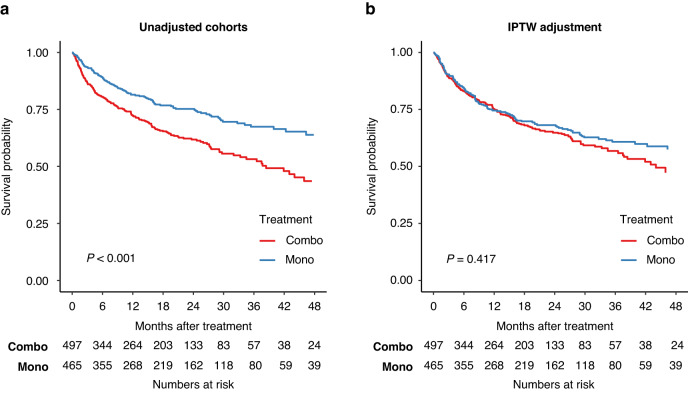
Overall survival stratified by treatment strategy. **a** Unadjusted and (**b**) IPTW adjusted Kaplan–Meier plot of the complete cohort by treatment.

To compare the effectiveness of combination and monotherapy we used OS, considering all-cause mortality, as the endpoint. Unadjusted Kaplan–Meier estimator suggested that monotherapy is associated with improved OS (Fig. 2a). This benefit of monotherapy, however, may be caused by the disparity between the two groups. We therefore performed propensity score adjustment using demographic and clinical covariates for weighting the Kaplan–Meier estimation. Following this adjustment, we did not observe a significant difference in survival between the treatments (HR: 0.90 [0.69–1.17], *P* = 0.417; Fig. 2b, Table 2). Since the BRAF mutational status was missing in about half of the patients, we next performed an analysis for each of the cohorts independently, which is also a sensitivity analysis. In the 483 patients with available BRAF status we added the mutational status as a covariate. In both analyses there was no significant difference between the treatment groups (With available BRAF status—HR: 0.89 [0.62–1.30], *P* = 0.56; Without available BRAF status—HR: 0.87 [0.60–1.25], *P* = 0.44; Supplementary Fig. 2).

**Table 2:** Expected survival stratified by treatment strategy.

		1-year	2-year	3-year
All	Monotherapy	0.75 [0.69–0.81]	0.68 [0.62–0.74]	0.61 [0.55–0.68]
	Combination therapy	0.75 [0.71–0.79]	0.65 [0.60–0.70]	0.57 [0.51–0.63]
Oligo-organ metastasis	Monotherapy	0.87 [0.83–0.91]	0.80 [0.75–0.85]	0.73 [0.67–0.79]
	Combination therapy	0.81 [0.77–0.85]	0.72 [0.66–0.77]	0.63 [0.56–0.70]
Multi-organ metastasis	Monotherapy	0.33 [0.18–0.48]	0.23 [0.10–0.35]	0.18 [0.06–0.29]
	Combination therapy	0.52 [0.43–0.62]	0.40 [0.30–0.50]	0.35 [0.25–0.46]

Survival levels are all after adjustment with IPTW.

We subsequently investigated whether a particular subgroup of patients might derive greater benefit from one treatment strategy over another. To this end, we employed a Cox proportional-hazards model, incorporating an interaction term between the treatment group and all clinical and demographic characteristics examined in this study. The only significant interaction observed within the treatment group was related to metastatic burden (*P* = 0.001; Supplementary Table 3). Additionally, we conducted Kaplan–Meier estimator analyses for subsets of the cohorts, considering each covariate, and adjusted for all other factors using propensity scores (Supplementary Fig. 3). Similarly, the sole statistically significant distinguishing factor was the metastatic burden.

**Fig. 3:**
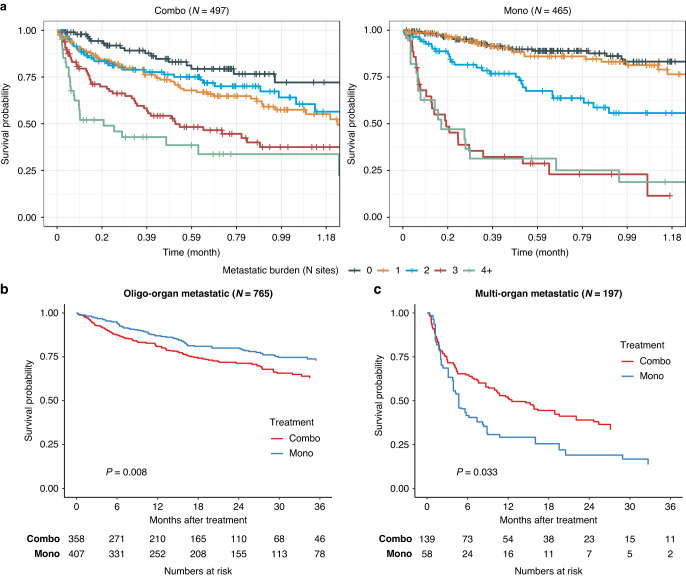
Number of organs involved is associated with overall survival. **a** Kaplan–Meier plots by number of involved organ systems for patients treated with combination therapy (left panel) or monotherapy (right panel). Adjusted Kaplan–Meier plot of patients with oligo-organ (**b**) and multi-organ (**c**) metastasis (number of involved organs).

Metastatic burden was defined as the number of distant organ systems involved, not including lymph node and unspecified types. The top involved organs were lung (*n* = 369; 38.4%), brain (*n* = 305; 31.7%), bone (*n* = 227; 23.6%) and liver (*n* = 230; 23.9%). In addition, 221 patients (23.0%) had involvement in less common distant organs, including digestive system, peritoneum, adrenal gland, intestine and other sites, which were all lumped together as “other sites”. Regardless of treatment strategy, we observed significant differences in survival between patients based on the number of organ systems involved (Fig. 3a). Importantly, for most patients (765; 79.5%) which suffered from 2 or less involved organ systems (hereafter, oligo-organ metastasis group), we observed a clear survival benefit for monotherapy (HR: 0.66 [0.48–0.90], *P* = 0.008); three-years survival rates in this subgroup were 73.1% and 63.2% for mono- and combination therapy, respectively (Fig. 3b; Table 2). In contrast, combination therapy provided benefit in OS for patients with 3 or more organ systems involved (hereafter, multi-organ metastasis group), as we observed a significant survival disadvantage for monotherapy in the 195 patients with multi-organ metastasis (HR: 1.67 [1.09–2.56], *P* = 0.033) three-years survival of 17.9% and 35.1% for mono- and combination therapy, respectively (Fig. 3c; Table 2). These significant associations with OS were maintained in both the oligo and multi-organ metastasis groups even when limiting the analysis to the subset of patients with available BRAF status (Supplementary Fig. 4).

### Therapy associated toxicity

We hypothesized that the lower OS benefit of the combination therapy in oligo-organ metastatic patients can be explained by the high toxicity attributed to this treatment. The difference between the two treatment groups is only in the first three months: in combination therapy, patients additionally receive the anti-CTLA-4 antibody ipilimumab, whereas thereafter all continue with PD-1 blockade alone. Therefore, we searched for hospitalization events in the first 90 days after the initial cycle of therapy and classified those hospitalizations as either irAE- or disease progression-related (Supplementary Data).

**Fig. 4:**
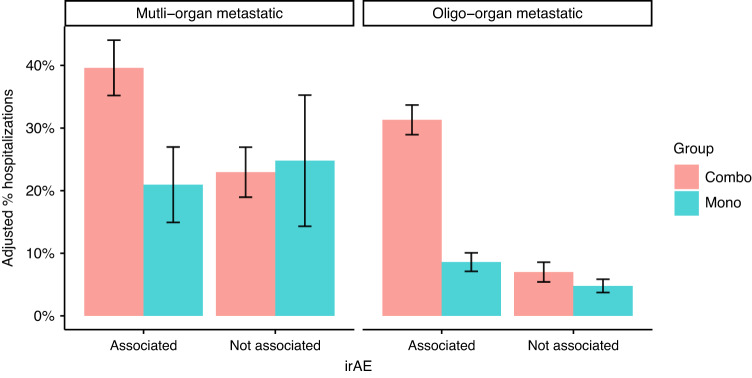
Posttreatment hospitalizations. Percent of patients with hospitalizations events treated with combination therapy (left panel) or monotherapy adjusted to all covariates using IPTW. Left: High metastatic burden patients (3 or more involved organs). Right: Oligo-organ metastasis patients (2 or less involved organs).

In the oligo-organ metastasis cohort we identified 193 patients (25.2%) who had a hospitalization event. Consistent with the literature, the combination therapy group had a hospitalization rate nearly three times higher than the other group, with 38.4% compared to 12.8% (*P* < 0.001). Notably, 81.2% of these additional hospitalizations were due to irAEs (Fig. 4). In the multi-organ cohort, hospitalization events were more prevalent (60.4%, *n* = 119). The combination therapy in this group saw a 1.4 times higher hospitalization rate, with 62.4% compared to 45.4% (*P* < 0.001), and all of these excess hospitalizations were associated with irAEs. Importantly, in contradiction to the clinical trials, irAE-associated hospitalizations did result in death: in the first 90 days of treatment, we found 17 patients who died up to two weeks after the hospitalization, 13 of them were treated with the combination and for 11 of them the hospitalization was designated as irAE-associated.

## Discussion

While the here reported real-world evidence supports the high clinical efficacy of ICI in advanced melanoma patients, it also suggests that the combination of PD-1- and CTLA-4- blocking antibodies, are mostly beneficial for patients with metastatic disease affecting more than two organ systems. This observation may be explained by the different characteristics of multi-metastatic and oligo-metastatic melanoma or by the fact that the potential benefit of combination therapy in patients with a low number of organs involved is outweighed by its higher toxicity.

Multi-metastatic melanomas exhibit greater heterogeneity or variability between different tumors than oligo-metastatic tumors [18]. This may be due to the need for cancer cells to adapt to different environments. On the other hand, oligo-metastatic melanoma tends to be associated with a stronger immune system response than multi-metastatic melanoma. Indeed, evasion of immune cells and the ability to enhance survival pathways, often achieved through interaction with the stroma, are essential for successful metastatic colonization.

The advantage of less intensive treatment for patients with oligo-metastatic disease is likely to be due to lower rates of therapy-associated toxicities, which appear to have a negative impact on survival, particularly during the first 3 months of treatment. However, on the long term, for patients who managed to survive the high toxicity, the combination may have some benefit. We argue that this may explain the marginal, non-significant, OS benefit for combination therapy at three-years in the CheckMate-067 trial [2], which became significant only at five years follow-up [3]. Our results are in line with a recent meta-analysis of randomized controlled trials in other cancer types, which showed that the combination therapy does not provide survival benefit [11].

### Strengths and limitations

This study has several strengths. First, our cohort of 962 patients is relatively large. Second, our data set includes a wide set of features that should be considered to match baseline differences across patients receiving different treatment regimens. Third, both the patient population as well as the treating oncologists are highly diverse coming from multiple clinical sites across the US. This diversity reduces the chances of selection bias in treatment decisions and increases the generalizability of the conclusions for patients with different background characteristics. On the other hand, it should be noted that the limitation of using only commercial insurance patients and the fact that all data is from the US, reduces the generalizability, and further studies should include other populations.

Our analysis also has several limitations. First, data were collected for administrative and reimbursement-related purposes. Thus, important features, which are commonly used in clinical practice were missing (e.g. PD-L1 expression on tumor cells) or available for only a subset of patients (BRAF mutational status). Additionally, performance-free survival (PFS) could not be examined as it cannot be reliably obtained from claims data. This omission is significant as overall survival (OS) is influenced by subsequent therapies. It should be noted that despite clinical trials demonstrating that combination therapy with ICIs often results in improved PFS, this does not invariably translate to enhanced OS. Another limitation of claims data is that coding practices may vary significantly across clinical sites and may not always be representative of the clinical procedures and thus the patient’s status; however, it should be noted that we addressed this problem by using a combination of claims and reported clinical data. Still, metastatic sites are not reported consistently, especially if no treatment can be offered to treat them. Nevertheless, our analysis identified a clear difference in survival according to the number of organs involved strengthening the relevance of the assignments for discriminating patients. Last, there are inherent limitations from time-related biases and other residual confounding from unmeasured factors. Finally, this was an exploratory analysis, to identify subsets of patients with differential response, and multiple hypotheses were tested, increasing the possibility to identify significant associations.

In summary, our real-world data suggest that intensified ICI by combining CTLA-4 and PD-1 blockade is particularly beneficial for patients with more than two involved organ systems whereas its theoretical benefit in patients with low number of involved organs is diminished due to its high toxicity. These findings should be verified using additional data sources to allow optimization of the current practice.

## Supplementary Information

Supplementary Tables & Figures

## Data Availability

Due to confidentiality and contractual requirements, supporting data cannot be made openly available. Additional information about the data used in this research, and requests to access it, can be made by application to the Elevance Health Digital Data Sandbox delivered by Carelon Digital Platforms Manager (datasandbox@anthem.com) at Carelon Digital Platforms (https://www.carelondigitalplatforms.com/).
