# Supplementary material for: Comparative efficacy of combined CTLA-4 and PD-1 blockade vs. PD-1 monotherapy in metastatic melanoma: a real-world study
Source: BJC Rep. 2024 Feb 13;2:14. doi: 10.1038/s44276-024-00041-1 (PMC11524072; doi:10.1038/s44276-024-00041-1)
Supplement: Supplementary file 1 — Supplementary Tables & Figures [file 44276_2024_41_MOESM1_ESM.docx]

# **Supplementary Tables & Figures**

**Supplementary Table 1. Characteristics of pembrolizumab and nivolumab treated patients.**

|  |  | **Nivolumab** | **Pembrolizumab** | **P-value** |
| --- | --- | --- | --- | --- |
| **N** |  | 225 | 240 |  |
| **Age [mean (SD)]** | | 56.71 (10.15) | 56.84 (11.26) | 0.414 |
| **Male sex (%)** | | 155 (68.9) | 136 (56.7) | 0.009 |
| **ECOG PS (%)** | 0 | 127 (56.4) | 133 (55.4) | 0.405 |
|  | 1 | 90 (40.0) | 92 (38.3) |  |
|  | 2 | 8 (3.6) | 15 (6.2) |  |
| **Treatment year (%)** | 2017 | 48 (21.3) | 64 (26.7) | 0.262 |
|  | 2018 | 52 (23.1) | 55 (22.9) |  |
|  | 2019 | 54 (24.0) | 41 (17.1) |  |
|  | 2020 | 34 (15.1) | 45 (18.8) |  |
|  | 2021 | 37 (16.4) | 35 (14.6) |  |
| **SDI (mean (SD))** ^$^ | | 36.30 (25.28) | 43.80 (26.04) | 0.002 |
| **CCI (mean (SD))** ^§^ | | 0.44 (0.74) | 0.48 (0.80) | 0.627 |
| **BRAF status**  **(%)** | MUT (%) | 59 (26.2) | 57 (23.8) | 0.611 |
|  | WT (%) | 66 (29.3) | 71 (29.6) |  |
| **Metastatic site**  **(%)** | Brain (%) | 53 (23.6) | 67 (27.9) | 0.333 |
|  | Lung (%) | 63 (28.0) | 89 (37.1) | 0.047 |
|  | Liver (%) | 36 (16.0) | 41 (17.1) | 0.85 |
|  | Bone (%) | 42 (18.7) | 47 (19.6) | 0.894 |
|  | Other sites (%) | 29 (12.9) | 48 (20.0) | 0.053 |
| **No. of metastatic sites (mean (SD))** | | 0.99 (1.10) | 1.22 (1.23) | 0.038 |

$ social deprivation index, § Charlson Comorbidity index

**Supplementary Table 2. Characteristics of treated patients stratified on BRAF status availability.**

| **BRAF status** |  | **Available** | **Unavailable** | **P-value** |
| --- | --- | --- | --- | --- |
| **N** |  | 483 | 479 |  |
| **Age [mean (SD)]** | | 54.88 (10.72) | 56.58 (10.46) | 0.013 |
| **Male sex (%)** | | 313 (64.8) | 313 (65.3) | 0.914 |
| **ECOG PS (%)** | 0 | 270 (55.9) | 248 (51.8) | 0.135 |
|  | 1 | 195 (40.4) | 201 (42.0) |  |
|  | 2 | 18 (3.7) | 30 (6.3) |  |
| **Treatment year (%)** | 2017 | 116 (24.0) | 102 (21.3) | 0.589 |
|  | 2018 | 90 (18.6) | 100 (20.9) |  |
|  | 2019 | 97 (20.1) | 84 (17.5) |  |
|  | 2020 | 88 (18.2) | 93 (19.4) |  |
|  | 2021 | 92 (19.0) | 100 (20.9) |  |
| **SDI (mean (SD)) $** | | 39.46 (25.73) | 40.88 (25.66) | 0.39 |
| **CCI (mean (SD)) §** | | 0.43 (0.73) | 0.59 (0.89) | 0.003 |
| **Metastatic site**  **(%)** | Brain (%) | 155 (32.1) | 150 (31.3) | 0.85 |
|  | Lung (%) | 193 (40.0) | 176 (36.7) | 0.337 |
|  | Liver (%) | 100 (20.7) | 130 (27.1) | 0.024 |
|  | Bone (%) | 118 (24.4) | 109 (22.8) | 0.592 |
|  | Other sites (%) | 105 (21.7) | 116 (24.2) | 0.403 |
| **No. of metastatic sites (mean (SD))** | | 1.39 (1.20) | 1.42 (1.30) | 0.687 |

$ social deprivation index, § Charlson Comorbidity index

**Supplementary Table 3.** Summary table of a Cox proportional-hazards model with interaction term between the treatment groups and the covariates used in this study.

|  | **Survival times** | | |
| --- | --- | --- | --- |
| *Predictors* | *Estimates* | *std. Error* | *CI* |
| Treatment (monotherapy) | 0.44 | 0.34 | 0.10 – 2.02 |
| gender [Male] | 0.94 | 0.15 | 0.68 – 1.29 |
| ECOG PS = 1 | 1.37 ^*^ | 0.22 | 1.01 – 1.87 |
| ECOG PS = 2 | 2.61 ^**^ | 0.82 | 1.41 – 4.82 |
| Year of treatment | 0.93 | 0.05 | 0.84 – 1.03 |
| Age | 1.00 | 0.01 | 0.99 – 1.01 |
| SDI | 1.01 ^*^ | 0.00 | 1.00 – 1.01 |
| CCI | 1.20 ^*^ | 0.10 | 1.01 – 1.42 |
| BRAF MUT | 0.43 ^***^ | 0.10 | 0.27 – 0.69 |
| BRAF WT | 0.66 ^*^ | 0.12 | 0.47 – 0.94 |
| # of metastatic sites | 1.40 ^***^ | 0.09 | 1.25 – 1.58 |
| Monotherapy × gender [M] | 0.73 | 0.19 | 0.44 – 1.21 |
| Monotherapy × PS=1 | 0.83 | 0.21 | 0.50 – 1.37 |
| Monotherapy × PS=2 | 0.71 | 0.33 | 0.29 – 1.75 |
| Monotherapy × year | 0.98 | 0.09 | 0.82 – 1.17 |
| Monotherapy × age | 1.01 | 0.01 | 0.98 – 1.03 |
| Monotherapy × SDI | 1.00 | 0.00 | 0.99 – 1.01 |
| Monotherapy × CCI | 1.14 | 0.15 | 0.87 – 1.49 |
| Monotherapy × BRAF MUT | 1.26 | 0.43 | 0.64 – 2.47 |
| Monotherapy × BRAF WT | 0.82 | 0.24 | 0.46 – 1.46 |
| Monotherapy × # of metastatic sites | 1.34 ^**^ | 0.12 | 1.12 – 1.61 |
| Observations | 962 | | |
| R^2^ Nagelkerke | 0.196 | | |
| ** p<0.05   ** p<0.01   *** p<0.001* | | | |

**Supplementary Figure 1. Survival curves of monotherapy treatments.** Adjusted Kaplan-Meier curves for patients treated with monotherapy, stratified by regimen (Nivo=nivolumab, Pembro=permbrolizumab).

**Supplementary Figure 2. Adjusted survival analysis for patients with BRAF status. A.** Analysis with 483 patients with available BRAF status. **B.** Analysis with 479 patients with unavailable BRAF status.

**Supplementary Figure 3. Kalan-Meier estimators of subsets of patients.**

**Supplementary Figure 4. Number of organs involved analysis limited to patients with BRAF status.** Similar figure as figure 3, but limited to 493 patients with available BRAF status. (**A)** Kaplan-Meier plots by number of involved organ systems for patients treated with combination therapy (left panel) or monotherapy (right panel) **(B and C)** Adjusted Kaplan-Meier plot of patients with oligo-organ (B) and multi-organ (C) metastasis (number of involved organs).

**Supplementary Data**

Definition of immune-related adverse event hospitalization was performed using ICD10 codes from Gunturu et al.^6^ and Wang et al.^15^. Following review of those lists we added an additional list of codes (see below) based on practice of Dr. Brenner. Since we have access to pharmacy codes, we also used codes that are indicative of treatment with steroids - prednisone, metylprednisolone and hydrocortisone.

If the hospitalization had at least two of the codes, or the code was a primary diagnosis, the hospitalization was marked as irAE-associated.

| ICD10 | Title |
| --- | --- |
| E035 | MYXEDEMA COMA |
| E039 | HYPOTHYROIDISM UNSPECIFIED |
| E064 | DRUG-INDUCED THYROIDITIS |
| E162 | HYPOGLYCEMIA UNSPECIFIED |
| E272 | ADDISONIAN CRISIS |
| E273 | DRUG-INDUCD ADRENOCORTICAL INSUFF |
| E2740 | UNS ADRENOCORTICAL INSUFFICIENCY |
| E278 | OTHER SPEC DISORDERS ADRENAL GLAND |
| G92 | TOXIC ENCEPHALOPATHY |
| G9341 | METABOLIC ENCEPHALOPATHY |
| I514 | MYOCARDITIS UNSPECIFIED |
| J189 | PNEUMONIA UNSPECIFIED ORGANISM |
| J9601 | ACUTE RESPIRATORY FAIL W/HYPOXIA |
| K521 | TOXIC GASTROENTERITIS AND COLITIS |
| K529 | NONINFECTIVE GE & COLITIS UNS |
| K7589 | OTH SPEC INFLAMM LIVER DISEASES |
| L308 | OTHER SPECIFIED DERMATITIS |
| N179 | ACUTE KIDNEY FAILURE UNSPECIFIED |
| R21 | RASH OTH NONSPECIFIC SKIN ERUPTION |
| R5381 | OTHER MALAISE |
| T380X5A | ADVRS EFF GLUCOCORT SYN ANALOG INIT |
| T451X5A | ADVRS EFF ANTINEOPL IMMUNOSUP INIT |
| Z7952 | LONG TERM USE OF SYSTEMIC STEROIDS |
